# Peroxisomal Targeting as a Sensitive Tool to Detect Protein-Small RNA Interactions through *in Vivo* Piggybacking

**DOI:** 10.3389/fpls.2018.00135

**Published:** 2018-02-09

**Authors:** Marco Incarbone, Christophe Ritzenthaler, Patrice Dunoyer

**Affiliations:** Institut de Biologie Moléculaire des Plantes du CNRS, UPR2357, Université de Strasbourg, Strasbourg, France

**Keywords:** small RNA, viral suppressor of RNA silencing, peroxisome, Arabidopsis, piggybacking

## Abstract

Peroxisomes are organelles that play key roles in eukaryotic metabolism. Their protein complement is entirely imported from the cytoplasm thanks to a unique pathway that is able to translocate folded proteins and protein complexes across the peroxisomal membrane. The import of molecules bound to a protein targeted to peroxisomes is an active process known as ‘piggybacking’ and we have recently shown that P15, a virus-encoded protein possessing a peroxisomal targeting sequence, is able to piggyback siRNAs into peroxisomes. Here, we extend this observation by analyzing the small RNA repertoire found in peroxisomes of P15-expressing plants. A direct comparison with the P15-associated small RNA retrieved during immunoprecipitation (IP) experiments, revealed that *in vivo* piggybacking coupled to peroxisome isolation could be a more sensitive means to determine the various small RNA species bound by a given protein. This increased sensitivity of peroxisome isolation as opposed to IP experiments was also striking when we analyzed the small RNA population bound by the *Tomato bushy stunt virus*-encoded P19, one of the best characterized viral suppressors of RNA silencing (VSR), artificially targeted to peroxisomes. These results support that peroxisomal targeting should be considered as a novel/alternative experimental approach to assess *in vivo* interactions that allows detection of labile binding events. The advantages and limitations of this approach are discussed.

## Introduction

Peroxisomes are small eukaryotic organelles that specialize in oxidative metabolic reactions (Kaur et al., 2009). Originating from the endoplasmic reticulum, they are surrounded by a single lipid membrane and typically measure 0.1 to 1 μm in diameter. The chemical reactions carried out within peroxisomes reflect their function and are determined by their enzymatic content, which in turn depends on species, cell type, and environmental conditions (Lanyon-Hogg et al., 2010). However, all peroxisomes perform the vital task of detoxifying reactive oxygen species. Plant peroxisomes play a pivotal role in a wide range of pathways such as lipid metabolism, photorespiration, nitrogen metabolism, hormone synthesis and plant-pathogen interactions (Hayashi and Nishimura, 2003; Kaur et al., 2009). Accordingly, mutations abolishing peroxisome biogenesis are embryo-lethal in *Arabidopsis thaliana* (Schumann et al., 2003; Fan et al., 2005).

The protein complement of plant peroxisomes is of 100+ proteins (Eubel et al., 2008; Reumann et al., 2009), roughly double that of mammalian peroxisomes (Wiese et al., 2007). Unlike mitochondria and chloroplasts, which encode and synthesize part of their protein complement, peroxisomes completely rely on protein import from the cytoplasm to the peroxisomal matrix. The process of protein import into peroxisomes is performed by a dedicated set of PEX proteins, collectively known as the importomer (Rayapuram and Subramani, 2006). The peroxisomal importomer is unique in its ability to import folded proteins and protein oligomers across the peroxisomal membrane and into the organellar matrix, in a phenomenon known as piggybacking (Leon et al., 2006; Rayapuram and Subramani, 2006; Lanyon-Hogg et al., 2010; Hasan et al., 2013). The process of protein import into peroxisomes has mostly been characterized using yeast and mammalian models, and can be divided into five stages (Hasan et al., 2013): (i) cargo recognition by the import receptor in the cytosol, (ii) docking of the receptor-cargo complex to the docking complex on the peroxisomal membrane, (iii) cargo translocation across the membrane into the peroxisomal matrix, (iv) release of the cargo, and (v) recycling of the import receptor to the cytoplasm for another round of import.

As in animals and fungi, plants have two import receptors, PEX5 and PEX7 (Kragler et al., 1998; Nito et al., 2002), which recognize peroxisomal targeting sequences PTS1 and PTS2, respectively. Most plant peroxisomal proteins possess a C-terminal PTS1 (Kaur et al., 2009), and are therefore imported by PEX5. The import process starts in the cytoplasm when PEX5 binds the PTS1 tripeptide present on the C-terminal end of its cargo protein, an event that has been shown to cause conformational changes in human PEX5 (Stanley et al., 2006). PEX5 and its cargo protein then approach and bind the docking complex present on the peroxisomal membrane, which is made up of PEX13 and PEX14 (Urquhart et al., 2000; Pires et al., 2003). The cargo is then translocated across the membrane, along with its co-factors or interactors, through a mechanism that remains elusive. Though several models have been proposed, the transient pore model can explain much of the data recovered on the subject (Erdmann and Schliebs, 2005; Hasan et al., 2013). Since PEX5 is able to bind lipids and behave like a membrane protein (Kerssen et al., 2006), this import receptor likely forms the pore along with PEX13 and PEX14. Interestingly, an *in vitro* reconstituted PEX5-PEX14 complex is able to act as a channel, with an opening of up to 9 nm, when incubated with PEX5-cargo complexes (Meinecke et al., 2010). After cargo release within the peroxisomal matrix, through a yet unclear mechanism, PEX5 is ubiquitinated (Kaur et al., 2013), extracted from the membrane (Platta et al., 2005) and deubiquitinated (Debelyy et al., 2011) before entering a new import cycle in the cytoplasm.

Small RNAs are 21-24nt-long RNA molecules generated by Dicer RNase III-like enzymes from various double-stranded RNA (dsRNA) precursors. They mediate a pan-eukaryotic regulation process known as RNA silencing, or RNA interference (RNAi), through their incorporation into Argonaute (AGO) proteins, where they act as sequence-specific guide to trigger either (i) cleavage or translational inhibition of the targeted RNA (Baumberger and Baulcombe, 2005; Brodersen et al., 2008; German et al., 2008), or (ii) DNA or histone modifications of the targeted DNA (Law and Jacobsen, 2010). In *A. thaliana*, four Dicer-like (DCL) proteins are responsible for small RNA biogenesis (Xie et al., 2004; Henderson et al., 2006). DCL1 processes endogenous stem-loop RNA structures into micro RNA (miRNA) of 21-22nt, which play key roles in regulating developmental genes (Mallory and Bouché, 2008; Voinnet, 2009). DCL3 mainly processes short PolIV (p4)/RDR2-derived dsRNA into 24nt small interfering (si)RNA, which mediate transcriptional gene silencing through the RNA-directed DNA methylation (RdDM) machinery (Cao et al., 2003; Chan et al., 2004; Xie et al., 2004; Blevins et al., 2015; Zhai et al., 2015). DCL3 can also process longer dsRNA derived from transgenes, endogenous inverted repeats and, on occasions, viruses (Xie et al., 2004; Dunoyer et al., 2007; Raja et al., 2010). Finally, DCL4 and DCL2 process long, perfectly or near-perfectly complementary, dsRNA into siRNA of 21 and 22nt, respectively. These long dsRNA can derive from viral RNA, transgenes, or discrete endogenous loci (Dunoyer et al., 2005, 2007; Blevins et al., 2006; Deleris et al., 2006; Henderson et al., 2006; Garcia-Ruiz et al., 2010; Wang et al., 2011). Of note, the DCL4/DCL2-dependent 21/22nt virus-derived siRNAs are the main effectors of the antiviral RNAi reaction, the major plant defense mechanism against phytoviruses.

Given the crucial role of RNA silencing during antiviral defense, viruses have, in turn, evolved proteins that block or hinder this process. These proteins, collectively known as viral suppressors of RNA silencing (VSRs), employ a variety of strategies to inhibit antiviral RNAi (Incarbone and Dunoyer, 2013). Among them, the most widespread strategy, used by VSRs such as the tombusviral P19 or the potyviral HC-Pro, is to bind and sequester 21-22nt siRNAs, thereby inhibiting their loading into AGO effectors and preventing an effective antiviral RNAi reaction (Vargason et al., 2003; Ye et al., 2003; Lakatos et al., 2004, 2006; Schott et al., 2012; Garcia-Ruiz et al., 2015). In most if not all cases, the ability of these VSRs to bind small RNAs *in vivo*, and the nature of these small RNAs, was assessed by immunoprecipitation (IP) experiments.

Similarly, the *Peanut clump virus* (PCV)-encoded P15, a PTS1-containing VSR, was recently found to bind small RNAs of 21-22nt in length, with an apparent higher affinity for the latter size *in vivo*. Interestingly, as a result of P15 import into peroxisomes, P15-bound antiviral siRNAs are piggybacked into these organelles in order to efficiently neutralize their spread to naïve tissues and promote PCV systemic infection (Incarbone et al., 2017). Intriguingly, whereas siRNAs were found readily associated to P15 through both IP and peroxisomal isolation, miRNAs were present in peroxisomes in a P15-dependent manner but were below detection level in P15 IP fractions (Incarbone et al., 2017; MI and PD unpublished observations).

These observations suggested that peroxisomal isolation could be used as a valuable alternative approach to the widely used IP experiments, that may provide more information about the VSR-associated cargoes. However, as these results were obtained with two different versions of P15 (wild-type P15 versus Flag-HA epitope-tagged P15), it was not possible to discriminate whether this difference reflected a true advantage of the peroxisomal isolation approach, or resulted from an altered binding ability of the epitope-tagged P15 used for IP experiments. Therefore, to conclusively address this question we decided to compare the two approaches, using the same tagged version of P15, targeted or not to peroxisomes. These experiments confirmed our initial observations and supported that peroxisomal isolation is a sensitive technique to identify labile or weakly interacting complexes formed *in vivo*. The idea that peroxisomes, within this experimental frame, can be used as containers, equipped with a unique importomer capable of molecular piggybacking, whose function is to accumulate a protein of interest and its interactors within a closed membrane, was further validated for another small RNA-binding VSR, TBSV-encoded P19. Although promising, the inability to efficiently target to peroxisomes AGO2, one of the two main plant antiviral AGO proteins, indicates, however, that this approach must be empirically tested for each candidate. The advantages and limitations of this novel approach of peroxisomal targeting are discussed.

## Materials and Methods

### Plant Material

35S:P15FHA^SKL^ construct was generated by amplifying P15FHA (Incarbone et al., 2017) with a reverse primer containing an SKL-encoding sequence (TCTAAACTG) before the stop codon, and cloning it through restriction (XmaI) and ligation into binary vector pCTL-35S. 35S:P19HA and 35S:P19HA^SKL^ were obtained similarly, except that the sequence encoding the HA tag was added to the reverse primer upstream of the stop codon (P19HA) or the SKL-encoding codons (P19HA^SKL^), and SalI/PstI restriction sites were used. 35S:AGO2 and 35S:AGO2^SKL^ were obtained by amplifying AGO2 (AT1G31280) genomic sequence from *A. thaliana* Col0, with primers containing attB sites for Gateway cloning (with SKL-encoding codons upstream of the stop in the case of AGO2^SKL^), inserted into pDONR221 through BP recombination, then into pH2GW7 binary vector through LR recombination. SUC-*SUL* (Himber et al., 2003; Dunoyer et al., 2005) and *ago2-1* (SALK_003380) plants were transformed through floral dip as previously described (Bechtold and Pelletier, 1998). Transformed lines were selected by growing them *in vitro* on MS medium containing hygromycin. The 35S:*P15FHA*/SUC:*SUL* line was previously described (Incarbone et al., 2017). All experiments were performed on 6–7-week old rosettes grown in growth chambers, with 12h/12h day/night cycles.

### Immunoprecipitation

HA-epitope IP was performed as previously described (Incarbone et al., 2017). 0.2 g of frozen rosette leaves were ground in liquid nitrogen in 1 ml lysis buffer (50 mM Tris-HCl pH 8, 150 mM NaCl, 1% Triton X-100, Roche Complete protease inhibitor cocktail) and incubated 15 min on a rotating wheel. After two clarifications (12000 *g* for 5 min at 4°C), an aliquot was set aside as input fraction, and 50 μl of anti-HA micro-beads (MACS system, Miltenyi Biotec, ref. 130-091-122) were added to the remaining lysate and incubated 30 min at 4°C on wheel. Next, the lysate containing the beads was deposited on a magnetic M column (Miltenyi Biotech) and allowed to flow through. An aliquot of the flow-through was kept for further analysis, and beads were washed by adding 2 × 500 μl of lysis buffer to the columns and allowed to pass through, then 100 μl of 20 mM Tris-HCl pH 7.5, after which excess liquid was removed. Beads were recovered in 1 ml of Tri-reagent (Sigma).

### Peroxisome Isolation

Peroxisome isolations were performed as previously described (Reumann and Singhal, 2014), with minor modifications. Two samples were treated in each experiment, and all procedures were performed at 4°C. Note that plants must not be frozen prior to peroxisome isolation. Before isolation, plants were kept in the dark for 16–20 h. After sampling tissue for total RNA/protein analysis and IP, 20 g of whole *A. thaliana* rosettes per sample were harvested and left on ice 2 h. Plant tissue was minced with a knife, then ground in 60 ml grinding buffer (170 mM Tricine pH 7.5, 1 M sucrose, 2 mM EDTA, 1% BSA, 10 mM KCl, 1 mM MgCl_2_, plus 0.5% PVP-40, 5 mM DTT and Roche Complete protease inhibitor cocktail added before use). The resulting pulp was filtered through Miracloth, the liquid divided into three tubes and centrifuged 1 min at 6700 *g* (Beckman rotor JA25.5). The clarified supernatant was pooled and deposited on 4 freshly prepared and chilled Percoll/sucrose gradients. These contain, from top to bottom, 3 ml of 15% Percoll (15% Percoll, 750 mM sucrose, 20 mM tricine, 1 mM EDTA, 0.2% BSA), 9 ml of 38% Percoll (38% Percoll, 750 mM sucrose, 20 mM tricine, 1 mM EDTA, 0.2% BSA), 2 ml of 2:1 mix 38% Percoll:36% sucrose, 2 ml of 1:2 mix 38% Percoll:36% sucrose, and 3 ml of 36% sucrose (36% sucrose w/w, 20 mM tricine, 1 mM EDTA). Gradients were centrifuged 12 min at 13200 *g*, then without stop 20 min at 27000 *g* (Beckman rotor JA25.5), with medium brake. Top layers were discarded, while the bottom 2–3 ml were kept, pooled and diluted up to a volume of 60 ml in 36% sucrose solution, divided in 3 tubes and centrifuged 30 min at 38700 *g* (Beckman rotor JA25.5). Next, 1 ml of the organellar phase on the bottom of each tube was directly harvested with a cut-tip pipette, transferred to a potter and gently homogenized. Then, the samples were deposited on a sucrose 41.2 to 60% discontinuous gradient (from top: 0.8 ml 41.2%, 1.6 ml 43.7%, 1.6 ml 46%, 2.4 ml 48.5%, 0.5 ml 50.5%, 1.6 ml 55.2%, 0.8 ml 60% sucrose w/w, 20 mM tricine, 1 mM EDTA). Gradients were ultra-centrifuged 40 min at 110800 *g* (Beckman rotor SW41), with maximum acceleration and brake. 1.5 ml of visible white peroxisome fraction within the 50.5% sucrose phase were harvested and frozen at –80°C.

### RNA Analysis

RNA extraction was performed using Tri-reagent (Sigma), according to manufacturer’s instructions. In the case of immunoprecipitated and peroxisomal RNA, 1.5 μl glycogen was added during isopropanol precipitation, which was allowed O/N at 4°C. Small RNAs were resolved through PAGE (low- or high-resolution gels, according to the experiment) and blotted as previously described (Dunoyer et al., 2007). RNA was chemically crosslinked on nylon membranes by incubating 1 h 30 min at 60°C on Whatmann paper imbibed with EDC solution, composed of 0.125 M 1-Methylimidazole (Sigma–Aldrich, ref. M50834) and 3% *N*-(3-Dimethylaminopropyl)-*N*′-ethylcarbodiimide hydrochloride powder (Sigma–Aldrich, ref. E7750) and 1% HCl 1 M. Detection of RNA species was achieved by hybridizing membranes in Sigma PerfectHyb^TM^ Plus buffer with PCR products labeled with α-^32^P-CTP through Klenow reaction (SUL, IR71) or with oligonucleotides labeled with γ^32^P-ATP through PNK reaction (U6, Rep2, ta-siRNA255, TAS3 5’D7(+), miR159, miR160, miR169, miR173, miR403, miR408; Probing order is available upon request). Hybridization was carried out O/N at 42°C, followed by 3x10 min washes in 2X SSC, 2% SDS at 50°C. Radioactive signal was revealed with autoradiographic films (Fujifilm). Stripping of the membrane before reprobing is done by submerging the membrane in 500 ml of boiling stripping buffer (0.1% SDS) for 3 × 10 min. The efficiency of the stripping was assessed by checking the membrane with a Geiger counter radiation detector.

### Protein Analysis

Total protein was obtained from frozen tissues through phenol extraction followed by methanol/ammonium acetate precipitation, as previously described (Hurkman and Tanaka, 1986). Peroxisomal and immunoprecipitated protein were obtained from the phenolic phase resulting from Tri-reagent RNA extraction, through precipitation in acetone, according to manufacturer’s instructions. Proteins were resolved by SDS-PAGE and electro-blotted onto Immobilion-P membrane (Millipore), which were then incubated with the appropriate antibody (@HA: Sigma–Aldrich ref. H6533; @HPR: Agrisera ref. AS11 1797; @P15: Incarbone et al., 2017; @P19: kindly provided by K. Bouarab; @AGO2: Garcia et al., 2012; @AGO1: Qi et al., 2005). After incubation with secondary antibody, membranes were revealed with Roche Lumilight Plus substrate (ref. 1201519600) and autoradiographic films (Fujifilm).

## Results

### Peroxisomal Targeting of P15FHA Reveals *in Vivo* Binding to 21nt miRNA

**FIGURE 1:**
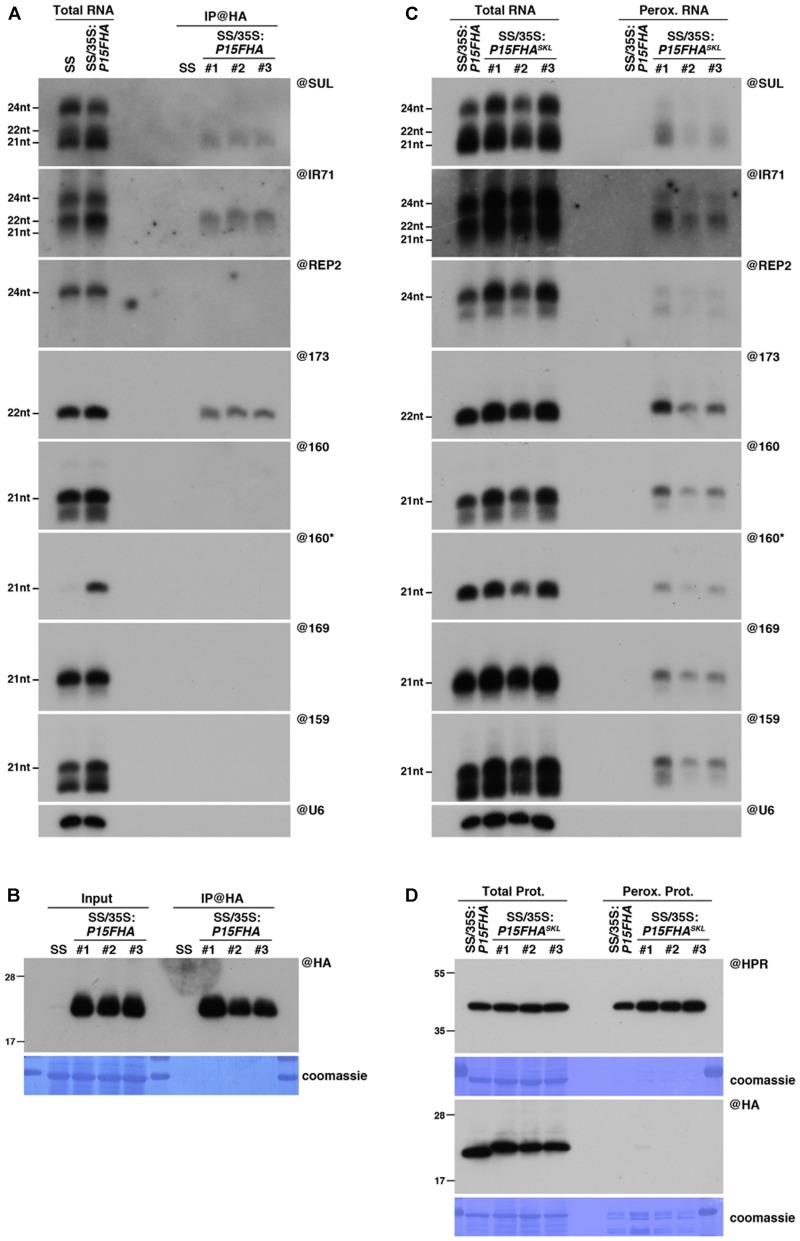
Peroxisomal targeting of P15FHA reveals P15/small RNA interactions not detected by immunoprecipitation. **(A)** Northern analysis of small RNAs (SUL, IR71, REP2, miR159, miR160, miR160^∗^, miR169, miR173) in total (left) and @HA immunoprecipitated (IP; right) fractions from SUC:*SUL* and 35S:*P15FHA*/SUC:*SUL* rosette leaves was obtained by sequential rounds of probing and stripping the same membrane. The three IP samples (#1, #2, #3) correspond to three technical replicates obtained from a pool of ten 35S:*P15FHA*/SUC:*SUL* plants. **(B)** Western analysis of P15FHA accumulation in total (left) and @HA IP (right) fractions obtained from the plants described in **(A)**. **(C)** Northern analysis of small RNAs in total (left) and peroxisomal (perox.; right) fractions obtained from 35S:*P15FHA*/SUC:*SUL* and 35S:*P15FHA^SKL^*/SUC:*SUL* plants. The small RNA species detected are the same as in **(A)**. The three peroxisome samples (#1, #2, #3) correspond to three biological replicates of 35S:*P15FHA^SKL^*/SUC:*SUL* plants. **(D)** Western analysis of plant peroxisomal marker hydroxypyruvate reductase (@HPR) and P15FHA/P15FHA^SKL^ (@HA) in total (left) and peroxisomal (right) fractions obtained from the plants described in (C). Note that more peroxisomal protein was loaded to detect P15FHA/P15FHA^SKL^ than to detect HPR. Northern analyses were performed on high-resolution gels. Accumulation of snU6 was used as RNA loading control, while coomassie staining was used as protein loading control. Figure source data can be found with the Supplementary information.

Previous experiments conducted with the VSR P15 have shown that although 21nt miRNAs could be detected in isolated peroxisomes, they were below detection level in northern analysis following IP experiments (Incarbone et al., 2017; MI and PD unpublished observations). These differences observed between IP and peroxisomal isolation approaches, regarding the detection of P15-bound miRNAs, could be a consequence of the different version of P15 used in these experiments. Indeed, whereas small RNA piggybacking in peroxisomes was assessed in wild-type P15-expressing plants, IP relied on a 2xFlag-2xHA epitope-tagged version of this VSR (P15FHA), which may exhibit an altered small RNA-binding ability compared to the untagged P15. Therefore, in order to compare side-by-side the sensitivity of the two approaches, we generated transgenic lines expressing the same epitope-tagged version of P15, fused or not in C-terminal to the canonical PTS1 tripeptide, serine (S)-lysine (K)-leucine (L) (P15FHA^SKL^ and P15FHA, respectively). Addition of this artificial PTS1 should allow to target P15FHA^SKL^ to peroxisomes, as opposed to the P15FHA-expressing lines where the tag prevents the peroxisomal import of P15 (Incarbone et al., 2017), by masking the PTS1 sequence naturally present in C-terminal of this VSR (Dunoyer et al., 2002). Both 35S:*P15FHA* and 35S:*P15FHA^SKL^* transgenes were introduced into the SUC:*SUL* reporter system, where an inverted-repeat (IR) construct, driven by the phloem-companion cell-specific AtSUC2 promoter, triggers RNAi of the endogenous *SULHUR* (*SUL*) mRNA and apparition of a chlorotic phenotype that expands 10-15 cells beyond the vasculature (Himber et al., 2003; Dunoyer et al., 2005). Of note, although the *SUL* IR is processed by DCL4 and DCL3 to generate 21nt and 24nt siRNAs, respectively, only the former is required for the appearance of the *SUL*-silencing phenotype (Dunoyer et al., 2007). We then selected a 35S:*P15FHA^SKL^*/SUC:*SUL* transgenic line that accumulated a similar amount of epitope-tagged P15 than the one found in our 35S:*P15FHA*/SUC:*SUL* line (**Figure 1D**), and producing similar levels of *SUL* siRNAs than the one found in our SUC:*SUL* reference line (**Figure 1C**).

Neither P15FHA- nor P15FHA^SKL^-expressing plants displayed a visible *SUL*-silencing phenotype, indicating that both proteins were equally competent in suppressing siRNA-mediated silencing (Supplementary Figure S1A). Accordingly, both proteins were able to bind 21nt SUL siRNAs, as revealed by their detection in both P15FHA IP fractions (**Figure 1A**) and P15FHA^SKL^ peroxisomal fractions (**Figure 1C**). Similarly, 22nt-long siRNAs deriving from the endogenous *IR71* locus, and the 22nt-long miRNA miR173, were found associated to P15 using both approaches (**Figures 1A,C**). However, the results were drastically different when we analyzed P15-binding to several 21nt-long miRNAs. Indeed, and in agreement with our previous report (Incarbone et al., 2017), all 21nt-long miRNAs tested were below detection level in P15FHA IP fractions (**Figure 1A**). In sharp contrast, all 21nt-long miRNAs were consistently found in three biological replicates of peroxisomal isolation performed on P15FHA^SKL^-expressing plants (**Figure 1C**). Given that these miRNAs were not detected in peroxisomal isolates of the P15FHA-expressing line, performed in parallel, this indicates that the miRNAs specifically detected in peroxisomes of plants expressing P15FHA^SKL^ result from P15-mediated piggybacking of these small RNAs. Moreover, detection in these peroxisomal isolates of the otherwise unstable miR160 passenger strand (miR160^∗^), strongly suggests that small RNAs are imported as duplexes by P15.

Collectively, these results suggest that, despite an apparent lower binding capacity towards 21nt miRNAs, P15 is able to associate, at least to a certain extent, with this specific class of small RNAs. This association is strong enough to cause the import of detectable levels of miRNA into peroxisomes, along with the other classes of P15-bound small RNAs. Once delivered into these organelles, their confinement within a closed membrane structure preserves them from the *ex vivo* treatment performed during peroxisomal isolation, whether or not they remain bound to P15, thereby allowing their detection. By contrast, P15 interaction with 21nt miRNAs seems to be lost upon *ex vivo* conditions applied during IP experiments (Incarbone et al., 2017). These observations prompted us to consider peroxisomal isolation as an alternative approach to the widely used IP experiments, that may provide more information about VSR-associated cargoes *in planta*.

Intriguingly, despite efficient P15FHA^SKL^-dependent piggybacking of small RNAs in peroxisomes (**Figure 1C**), the P15FHA^SKL^ protein was difficult to detect within peroxisomal isolates, using either antibodies raised against the HA tag or the P15 protein (**Figure 1D** and Supplementary Figure S1B). This was surprising given that (i) both hydroxypyruvate reductase (HPR), our peroxisomal marker, and wild-type untagged P15 were readily detectable in, respectively, this and previous analyses of peroxisomal fractions (Incarbone et al., 2017), and that (ii) P15FHA^SKL^ was found to accumulate to high levels in total protein extracts (**Figure 1D** and Supplementary Figure S1B). These observations suggest that either the P15FHA^SKL^ is quickly recycled to the cytoplasm following peroxisomal import and release of the bound small RNAs, and/or that addition of the 2xFlag2xHA tag to P15 triggers the prompt degradation of the P15FHA^SKL^ within peroxisomes by a currently unknown mechanism.

### Addition of a PTS1 Peptide to P19 Allows Efficient Piggybacking of Small RNAs into Peroxisomes

The more sensitive detection of P15-bound small RNAs in peroxisomal fractions compared to IP experiments (**Figures 1A,C**) prompted us to determine whether these observations were specific to P15, or if this trend could also be observed with another, unrelated, small RNA-binding VSR upon artificial targeting to peroxisomes. For this purpose, we decided to test the tombusviral P19. P19 has been extensively studied in the past and is potentially the best characterized VSR to date. The structure of P19 has been resolved and showed that a head-to-tail P19 homodimer binds preferentially 21nt-long small RNA duplexes with 2nt 3’ overhangs (Vargason et al., 2003; Ye et al., 2003). Accordingly, IP experiments revealed that P19 efficiently binds and sequesters 21nt siRNAs and miRNAs (Chapman et al., 2004; Schott et al., 2012), thereby preventing their loading into AGO effectors (Schott et al., 2012; Kontra et al., 2016; Incarbone et al., 2017).

**FIGURE 2:**
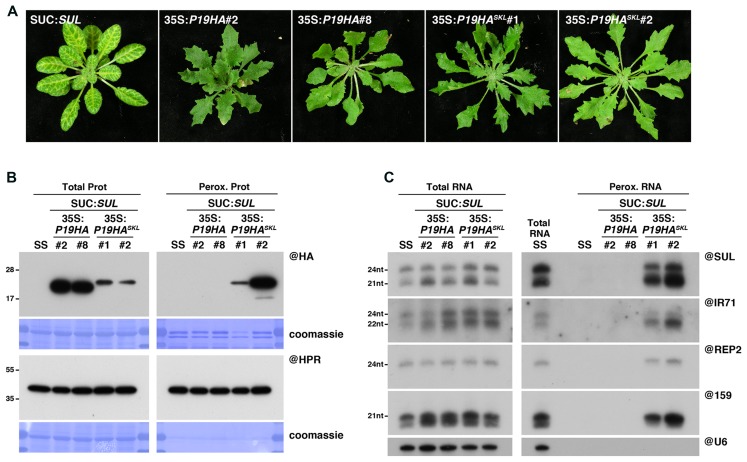
Peroxisomal targeting of P19HA leads to import of 21, 22, and 24nt small RNAs into these organelles. **(A)** Photos of SUC:*SUL*, 35S:*P19HA*/SUC:*SUL* (transgenic lines #2 and #8) and 35S:*P19HA^SKL^*/SUC:*SUL* (transgenic lines #1 and #2) plants used for peroxisome isolation. **(B)** Western analysis of plant peroxisomal marker hydroxypyruvate reductase (@HPR) and P19HA/P19HA^SKL^ (@HA) in total (left) and peroxisomal (perox. right) fractions obtained from the plants described in **(A)**. Note that more peroxisomal protein was loaded to detect P19HA/P19HA^SKL^ than to detect HPR. **(C)** Northern analysis of small RNAs (SUL, IR71, REP2, miR159) in total (left) and peroxisomal (right) fractions from the plants described in **(A)** was obtained by sequential rounds of probing and stripping the same membranes. Northern analysis was performed on a low-resolution gel. Accumulation of snU6 was used as RNA loading control, while coomassie staining was used as protein loading control. Figure source data can be found with the Supplementary Information.

In order to directly compare the results of IP versus peroxisomal isolation regarding detection of P19-bound small RNAs, we first generated a peroxisome import-competent version of P19, using the same rationale as for P15. Thus, we generated SUC:*SUL* transgenic lines expressing an HA-tagged version of P19, fused or not in C-terminal to the canonical PTS1 tripeptide, SKL (35S:*P19HA^SKL^*/SUC:*SUL* or 35S:*P19HA*/SUC:*SUL*, respectively). Western blot analysis revealed that, despite lower accumulation of P19HA^SKL^ than P19HA in total fractions, only the former was detected in peroxisomal isolates (**Figure 2B**), supporting functional targeting of this protein to peroxisomes. Importantly, both constructs efficiently impaired the appearance of the SUL-silencing phenotype (**Figure 2A**), without affecting production of SUL siRNAs (**Figure 2C**), indicating that addition of the SKL tripeptide did not hinder P19 ability to suppress siRNA-mediated silencing. Moreover, expression of P19HA^SKL^ triggered the appearance of similar developmental defects (mostly characterized by leaf serrations) as the one observed in P19HA-expressing lines (**Figure 2A**). Given that this phenotype has been previously associated with a disruption of the miRNA pathway (Kasschau et al., 2003; Chapman et al., 2004; Jay et al., 2011), these observations suggest that both proteins were also efficient in suppressing miRNA-mediated silencing.

We next wondered whether addition of a PTS1 to the tombusviral P19 also leads to piggybacking of P19-bound small RNA into peroxisomes. In agreement with the absence of the *SUL*-silencing phenotype, the presence of developmental defects (**Figure 2A**), and the documented size-specificity of P19 (Vargason et al., 2003; Ye et al., 2003; Lakatos et al., 2006; Mérai et al., 2006), northern analysis revealed that both 21nt SUL siRNAs and miR159 were indeed readily detected in peroxisomal isolates of P19HA^SKL^-expressing plants but not in control plants (**Figure 2C**), indicating specific P19-mediated piggybacking of these small RNAs into peroxisomes. More intriguingly, DCL3-dependent 24nt long siRNAs deriving from the exogenous *SUL*-IR transgene, the endogenous *IR71* locus or the p4-siRNA *REP2* loci, were also specifically detected in these fractions, suggesting that this small RNA size class can also be imported into peroxisomes along with P19HA^SKL^. Although surprising given that (i) *in vitro* binding assays showed that P19 has a 22-fold lower affinity for 24nt siRNA than for its 21nt counterpart (Vargason et al., 2003), and (ii) that 24nt SUL siRNAs were not detected in previous P19 IP experiments (Incarbone et al., 2017), these results may further support the advantage of the peroxisomal isolation approach to detect unstable or labile interactions, compared to IP experiments.

### Peroxisomal Targeting of P19 Reveals *in Vivo* Binding to DCL3-Dependent 24nt siRNA

**FIGURE 3:**
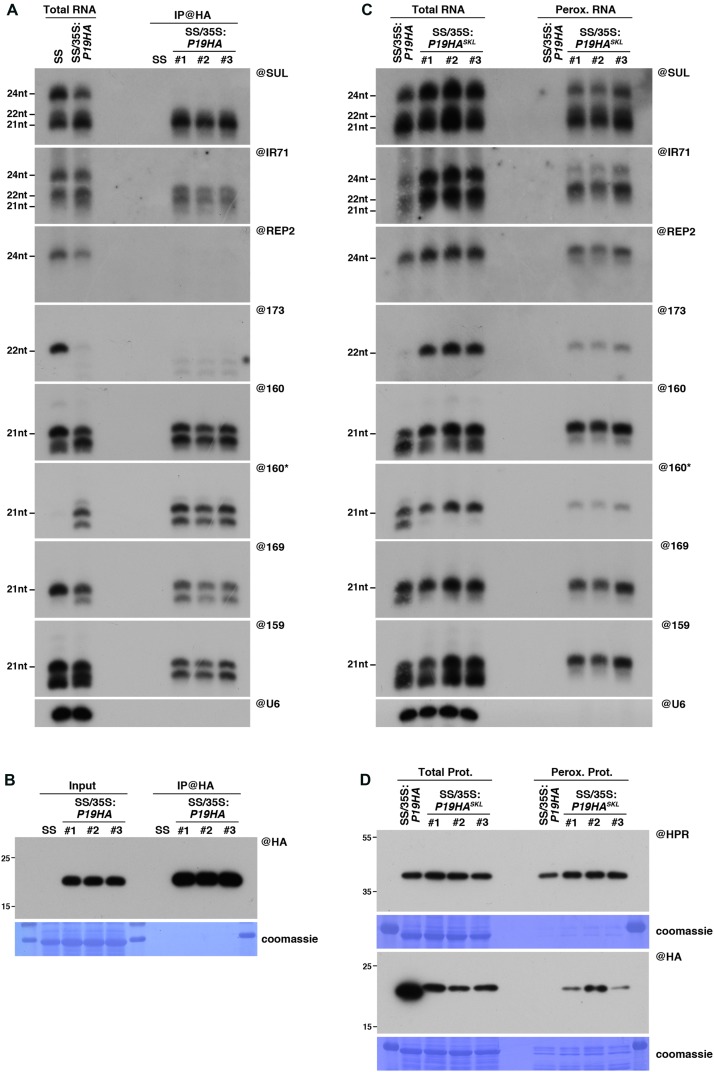
Peroxisomal targeting of P19HA reveals P19/small RNA interactions not detected by immunoprecipitation. **(A)** Northern analysis of small RNAs (SUL, IR71, REP2, miR159, miR160, miR160^∗^, miR169, miR173) in total (left) and @HA immunoprecipitated (IP; right) fractions from SUC:*SUL* and 35S:*P19HA*/SUC:*SUL* rosette leaves was obtained by sequential rounds of probing and stripping the same membrane. The three IP samples (#1, #2, #3) correspond to three technical replicates obtained from a pool of ten 35S:*P19HA*/SUC:*SUL* plants. **(B)** Western analysis of P19HA accumulation in total (left) and @HA IP (right) fractions obtained from the plants described in **(A)**. **(C)** Northern analysis of small RNAs in total (left) and peroxisomal (perox.; right) fractions obtained from 35S:*P19HA*/SUC:*SUL* and 35S:*P19HA^SKL^*/SUC:*SUL* plants. The small RNA species detected are the same as in **(A)**. The three peroxisome samples (#1, #2, #3) correspond to three biological replicates of 35S:*P19HA^SKL^*/SUC:*SUL* plants. **(D)** Western analysis of plant peroxisomal marker hydroxypyruvate reductase (@HPR) and P19HA/P19HA^SKL^ (@HA) in total (left) and peroxisomal (right) fractions obtained from the plants described in **(C)**. Note that more peroxisomal protein was loaded to detect P19HA/P19HA^SKL^ than to detect HPR. Northern analyses were performed on high-resolution gels. Accumulation of snU6 was used as RNA loading control, while coomassie staining was used as protein loading control. Figure source data can be found with the Supplementary information.

To confirm these observations, we decided to perform a side-by-side comparison of the P19-bound small RNAs retrieved using these two approaches. Similarly, to the experiments described above in the case of P15 (**Figure 1**), both IP and peroxisome isolations were performed in triplicate from P19HA- or P19HA^SKL^-expressing plants, grown in parallel in the same conditions and harvested simultaneously. In agreement with previous reports, IP experiments showed that, out of the 21nt and 24nt *SUL*-siRNAs produced in these plants, P19HA only efficiently binds to the former species (**Figure 3A**). In addition, analysis of the endogenous IR71-derived siRNAs, which are produced by DCL2, DCL3 and, to a lower extent, by DCL4, revealed that both 21nt and 22nt siRNAs can be immunoprecipitated by P19HA, although with an apparent bias toward the 21nt species (**Figure 3A**). Importantly, neither the *SUL*-, *IR71*-, nor *REP2*-derived 24nt siRNAs were found associated to P19HA in these IP experiments.

Similarly, to the 21nt siRNAs, 21nt miRNAs were also efficiently immunoprecipitated by P19HA, together with 1 or 2 nucleotide-shorter derivatives (**Figure 3A**), which most likely correspond to the recently described 3′-end trimming products of P19-bound miRNAs (Kontra et al., 2016). A similar trimming product was also observed for the miR160^∗^ passenger strand in our IP fractions, indicating that both 2nt 3’ overhang extremities of the P19-bound miRNA duplexes can be attacked by the exonuclease activity responsible for this shortening. As previously observed (Moissiard et al., 2007; Incarbone et al., 2017), expression of P19 triggers a strong decrease in miR173 accumulation (**Figure 3A**). Although the mechanism behind this specific destabilization remains unknown, detection with the miR173 probe of several shorter bands in both total and IP fractions suggests that this specific miRNA duplex may be particularly sensitive to the P19-induced 3′end trimming enzymatic activity.

In parallel, the results obtained from northern analysis of the small RNAs retrieved in our peroxisomal isolates (**Figure 3C**) yielded interesting differences compared to the IP experiments. First, in addition to the 21nt and 22nt siRNA species, both *SUL*- and *IR71*-derived 24nt siRNAs were readily detected in peroxisomes of P19HA^SKL^-expressing plants, as were the *REP2*-derived 24nt siRNAs (**Figure 3C**). Importantly, none of these siRNA species were detected in peroxisomal isolates of P19HA-expressing plants performed in parallel, supporting that their detection in peroxisomes of P19HA^SKL^-expressing plants specifically results from their piggybacking into these organelles by the P19HA^SKL^. In addition, western and mass spectrometry analysis of these peroxisomal extracts revealed that presence of these small RNA species in peroxisomes cannot be attributed to import of AGO proteins or other known silencing factors within these organelles upon P19HA^SKL^ expression (Supplementary Figure S2A and Supplementary Table S1). This indicates that P19 is able to bind 24nt siRNA species *in vivo*, but that this interaction is most likely lost during the *ex vivo* treatment applied during IP experiments, while it is maintained throughout the import into peroxisomes. Secondly, although miRNAs were, as expected, found to be efficiently imported into peroxisomes of P19HA^SKL^-expressing plants, we observed a strong reduction in the accumulation of their 1-2nt-shorter derivatives in both total and peroxisomal RNA fractions (**Figure 3C**), suggesting that peroxisomal import of P19-bound miRNAs protects them from the 3′-end trimming activity present in the cytoplasm. Consequently, miR173 accumulation, which was at or below detection level in P19HA-expressing plants, is restored to wild-type levels in the presence of P19HA^SKL^. Therefore, both P15 and P19 experiments (**Figures 1**, **3**) support peroxisomal isolation as an alternative approach to obtain more information about VSR-associated small RNAs *in planta* than that provided by conventional IP experiments.

Of note, and while not as drastically different as what we observed above in the case of P15FHA^SKL^ (**Figure 1D**), detection of the P19HA^SKL^ within peroxisomal isolates was also difficult to achieve using both anti-HA or anti-P19 antibodies, and required the loading of a higher amount of peroxisomal protein than the one required to detect HPR (**Figure 3D** and Supplementary Figure S2B). Although beyond the scope of this paper, these observations suggest that addition of an HA tag (one for P19HA^SKL^ and two for P15FHA^SKL^) seems to promote degradation of the tagged protein within peroxisomes, or their prompt export, by an unknown mechanism.

### Addition of a PTS1 to AGO2 Does Not Lead to Its Import into Peroxisomes

**FIGURE 4:**
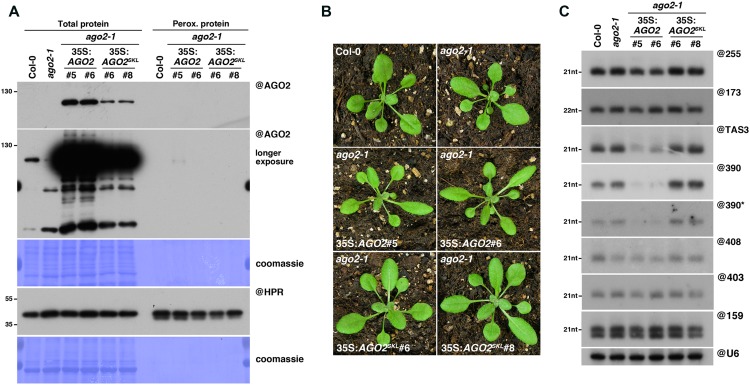
AGO2^SKL^ is not imported into peroxisomes, while overexpression of AGO2 impacts the miR390/*TAS3* pathway. **(A)** Western analysis of plant peroxisomal marker hydroxypyruvate reductase (@HPR) and AGO2/AGO^SKL^ (@AGO2) in total (left) and peroxisome (right) fractions obtained from Col0, 35S:AGO2/*ago2-1* (transgenic lines #5 and #6) and 35S:AGO2^SKL^/*ago2-1* (transgenic lines #6 and #8) plants. Total proteins also include *ago2-1*. **(B)** Photos of the plants described in **(A)**. **(C)** Northern analysis of small RNAs (miR159, miR173, miR390, miR390^∗^, miR403, miR408, *TAS1*-derived siRNA (255), and *TAS3*-derived siRNA 5′D7(+) (TAS3)) in total leaf tissue of plants described in **(A,B)** was obtained by sequential rounds of probing and stripping the same membrane. Northern analysis was performed on a low-resolution gel. Accumulation of snU6 was used as RNA loading control, while coomassie staining was used as protein loading control. Figure source data can be found with the Supplementary Information.

We next decided to assess whether peroxisomal targeting can also be used to determine RNA or protein interactors of the RNA silencing effector AGO2, which plays key roles during the antiviral silencing reaction against several RNA viruses (Harvey et al., 2011; Jaubert et al., 2011; Wang et al., 2011; Carbonell et al., 2012; Garcia-Ruiz et al., 2015). For this purpose, we generated, in an *ago2-1* mutant background, transgenic lines expressing AGO2, fused or not in C-terminal to the canonical PTS1 tripeptide SKL (35S:*AGO2^SKL^*/*ago2-1* or 35S:*AGO2*/*ago2-1*, respectively). Unfortunately, despite high levels of AGO2^SKL^ accumulation in total fractions, we were unable to detect any import of this protein in peroxisomal fractions (**Figure 4A**), suggesting that addition of a C-terminal PTS1 does not lead to peroxisomal import of AGO2. While the reason for this lack of AGO2 import into peroxisomes remains to be formally determined, we believe this is most likely caused by the inaccessibility of the PTS1 signal to PEX5, as crystal structures of human and yeast Argonaute proteins have shown that their C-terminal ends are not exposed on the surface, but rather buried within the RNA binding cleft (Poulsen et al., 2013).

Interestingly, while 35S:*AGO2^SKL^*-expressing plants were phenotypically undistinguishable from wild-type plants, we noticed that our 35S:*AGO2*/*ago2-1* transgenic lines displayed an elongated, “zippy” leaf phenotype (**Figure 4B**), typically observed in plants deficient for *TAS3* trans-acting (ta)-siRNA production (Adenot et al., 2006; Garcia et al., 2006). The production of *TAS3* ta-siRNAs relies on a unique two-hit pathway involving the loading of miR390 into AGO7, cleavage of the *TAS3* transcript in one of the two AGO7-miR390 binding sites, conversion by RDR6 of the cleaved TAS3 transcript into dsRNA and its subsequent dicing by DCL4 into ta-siRNAs (Axtell et al., 2006; Montgomery et al., 2008). However, miR390 has also been reported to be loaded into AGO2 (Montgomery et al., 2008; Takeda et al., 2008; Fatyol et al., 2015), possibly because of its 5′A, which has been shown to promote small RNA loading into this Argonaute (Mi et al., 2008).

In agreement with their elongated leaf phenotype, northern analysis revealed that the TAS3 5’D7(+) ta-siRNA accumulation was indeed strongly reduced in our 35S:*AGO2* transgenic lines (**Figure 4C**). We hypothesized that this reduction could be a consequence of direct competition for miR390 loading between AGO7 and AGO2. This could result from the overexpression of AGO2, assuming that the AGO2/miR390 complex would be less efficient than the AGO7/miR390 complex to trigger TAS3 ta-siRNA production. Surprisingly, we found that miR390 steady-state level was also strongly reduced in our 35S:*AGO2* transgenic lines, whereas the other miRNAs tested accumulated to wild-type levels (**Figure 4C**). As mutants impaired in TAS3 ta-siRNA production, such as *rdr6*, are not affected in miR390 accumulation (Fahlgren et al., 2006), this indicates a specific effect of AGO2 overexpression. Given that upon loading of the miRNA guide strand into AGO proteins, the complementary miRNA passenger strand (miR^∗^) is rapidly degraded, we next hypothesized that AGO2 in our transgenic lines might be preferentially loaded with miR390^∗^, thereby triggering degradation of the miR390 guide strand. The corollary of this hypothesis is that the miR390^∗^ should be stabilized in AGO2 over-expressing compared to wild-type plants, as previously observed in the case of miR393^∗^ loading into AGO2 during *Pseudomonas syringae* infection (Zhang et al., 2011). However, northern analysis revealed a strong reduction of miR390^∗^ in our 35S:*AGO2* transgenic plants, leaving open the molecular mechanism behind these observations.

## Discussion

The experiments described in this paper have shown that the addition of a PTS1 peptide to two distinct VSRs led to the piggybacking of their bound small RNAs into the peroxisomal matrix. More importantly, we found that these proteins were able to piggyback into peroxisomes small RNA species that were not detected following IP experiments performed in parallel (**Figures 1**, **3**). Therefore, these results delineate the potential of peroxisomal targeting as a tool to probe more accurately the small RNA interactome of VSRs *in vivo*. We believe that the increased sensitivity of this approach, when compared to IP experiments, mainly results from three combined factors. Firstly, while during IP, interactions must withstand *ex vivo* conditions for a considerable amount of time (from sample grinding to washing of the IPed immune complex) before RNA extraction and analysis, peroxisomal targeting and isolation allows to detect an interaction the moment a VSR-bound small RNA enters the peroxisome *in vivo*. Indeed, these organelles will work as small containers that will preserve the small RNA enclosed within the peroxisomal membrane from the *ex vivo* conditions applied during the peroxisome isolation procedure, whether they remain bound to the VSR or not. Secondly, although the fate of small RNAs piggybacked into peroxisomes remains an open question, their easy detection supports that a considerable amount remains intact. Moreover, as evidenced by the protection of P19-bound small RNAs from the 3′-end trimming activity present in the cytoplasm (**Figure 3**), another advantage of this peroxisomal targeting strategy is to protect/shelter VSR interactors from *in vivo* factors that may affect their nature or stability. Thirdly, while the small RNA co-IPed with a VSR are strictly the ones associated at the time of tissue harvest, the small RNA present in peroxisomes are most likely the result of VSR-dependent piggybacking during a certain period of time, spanning the life (or the period of importomer function) of each peroxisome. Therefore, interactors that are below detection level after IP could, thanks to their accumulation within peroxisomes, be detected by peroxisomal piggybacking.

Efficient detection of 24nt siRNAs in peroxisomes of P19HA^SKL^-expressing plants is in line with this latter point (**Figures 2**, **3**). Indeed, structural data combined with *in vitro* binding assays clearly established that P19 dimers form a molecular caliper that specifically accommodate 21nt-long small RNA duplexes for which they have the strongest affinity (Vargason et al., 2003; Ye et al., 2003). Moreover, the absence of 24nt siRNAs, as opposed to the clear detection of 21nt siRNAs, in several independent P19 IP experiments further supported the high P19 specificity for RNA duplexes of this latter size class (Lakatos et al., 2004; Kontra et al., 2016; Incarbone et al., 2017). Our results, however, support that 24nt siRNAs are significantly bound by P19 *in vivo*, possibly in a bent or deformed conformation. This interaction is most likely too weak or labile to withstand *ex vivo* conditions and P19-bound 24nt siRNAs are lost during IP procedures, whereas they accumulate within peroxisomes when P19 is targeted to these organelles *in vivo*.

Piggybacking into peroxisomes of DCL3-dependent 24nt siRNAs is somewhat surprising given that DCL3 and other RNA-directed DNA methylation (RdDM) factors, involved in the biogenesis and action of p4-siRNAs such as REP2, have been described as being exclusively located in the nucleus (Xie et al., 2004; Pontes et al., 2006; Law and Jacobsen, 2010), whereas P19 is mostly cytoplasmic (Papp et al., 2003). So how are DCL3-dependent siRNAs piggybacked into peroxisomes by P19HA^SKL^? We propose two alternative scenarios to explain this observation. The first one involves diffusion of P19HA^SKL^ into the nucleus, binding to the DCL3-dependent siRNAs and exit from the nucleus, prior to its recognition by PEX5. The second one relies on the previously described cytoplasmic step of the RdDM pathway (Ye et al., 2012). In this study, the authors showed that, following their biogenesis in the nucleus, p4-siRNA duplexes are exported into the cytoplasm where they are loaded into AGO4, the main RdDM effector in plants. Upon loading, AGO4-mediated cleavage triggers the removal of the passenger strand resulting in formation of mature AGO4/p4-siRNA complexes, which are in turn imported into the nucleus where they act (Ye et al., 2012). Therefore, the observed piggybacking of 24nt siRNAs by the peroxisomal-targeted P19 may involve their binding by P19HA^SKL^ in the cytoplasm, after their exit from the nucleus but prior to their loading into AGO4. Irrespectively of the way P19 binds 24nt siRNAs, it will be interesting to assess whether the amount of p4-siRNAs sequestered by P19 is sufficient to trigger a visible effect on AGO4 loading and RdDM.

Although promising in terms of accurate characterization, or even discovery, of potential interactors for a given protein, the peroxisomal targeting approach also entails several technical or biological limitations. From a practical point of view, peroxisome isolation is a longer and more technically challenging procedure than IP. It also requires an important amount of plant material and cannot be performed on frozen tissues. A second potential issue, as suggested by the apparent failure to import AGO2 in peroxisomes (**Figure 4**), is that the PTS1 tripeptide must be accessible for recognition by PEX5 and that, as opposed to tag fusion for IP experiments, this targeting signal must be on the C-terminal end of the studied protein. An alternative to this latter limitation could be the use of an N-terminal fusion sequence containing the PTS2 nonapeptide (Reumann, 2004) that can also drive peroxisomal localization after recognition by the cytoplasmic receptor PEX7 (Marzioch et al., 1994; Rehling et al., 1996), but this remains to be tested. A third foreseeable limitation is that this approach is less likely to yield meaningful results for proteins not acting in the cytoplasm, as their recognition by the cytoplasmic receptor PEX5 could prevent their localization to the appropriate subcellular compartment.

In addition to these points, there are other potential issues that cannot be predicted *a priori*. Firstly, recognition and binding to PEX5 of the peroxisomal-targeted protein of interest must not disrupt its association with its interactors. Secondly, the protein of interest and its interactors must be successfully shuttled onto the docking complex and pass through the peroxisomal pore, which may not be possible for large protein complexes due to potential size/conformation restrictions. Finally, once delivered within peroxisomes, the interactors must remain stable and not be degraded by peroxisomal enzymes. Because of these actual or potential limitations, this experimental approach must therefore be tested empirically for each candidate protein, in a case-by-case manner. However, we believe that this conceptually new approach of using peroxisomes as storage units that rely on cell machinery-driven accumulation of interactors within closed membranes, holds interesting promise for the full characterization of any given protein, particularly for the identification of RNA or protein components that are only weakly or transiently associated to it. Combining this approach with a more sensitive detection procedure such as high-throughput sequencing technologies should really be considered in the future in order to obtain a genome-wide view and full-spectra of the RNAs potentially bound by the protein of interest.

## Author Contributions

MI and PD designed and planned the experiments. MI performed the experiments. MI, CR, and PD analyzed the data. MI drafted the manuscript. CR and PD reviewed/revised the manuscript. All authors approved the final version to be published.

## Conflict of Interest Statement

The authors declare that the research was conducted in the absence of any commercial or financial relationships that could be construed as a potential conflict of interest.
