# Supplementary material for: Peroxisomal Targeting as a Sensitive Tool to Detect Protein-Small RNA Interactions through in Vivo Piggybacking
Source: Front Plant Sci. 2018 Feb 9;9:135. doi: 10.3389/fpls.2018.00135 (PMC5812032; doi:10.3389/fpls.2018.00135)

frontiers

Supplementary Material

Peroxisomal targeting as a sensitive tool to detect protein-small RNA interactions through *in vivo* piggybacking

Marco Incarbone[1], Christophe Ritzenthaler[1] and Patrice Dunoyer[1]*

*** Correspondence:** Patrice Dunoyer: Patrice.dunoyer@ibmp-cnrs.unistra.fr

Supplementary Figures:

Supplementary Figure 1: (**A**) Photos of SUC:*SUL,* 35S:*P15FHA*/SUC:*SUL* and 35S:*P15FHA*^{SKL}/SUC:*SUL* plants used for peroxisome isolation. Note the SUC:*SUL* plant depicted here is the same as in Figure 2A as all these plants were grown in parallel. (**B**) Additional exposure of the @HA western blot shown in Figure 1D, and two different exposures of a western blot performed on the same samples using an @P15 antibody. Note that the @HPR blot and the first @HA exposure are the same as in Figure 1D. Coomassie staining was used as protein loading control. Figure source data can be found with the Supplementary information.

Supplementary Figure 1

A

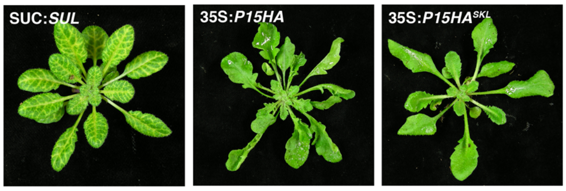

B

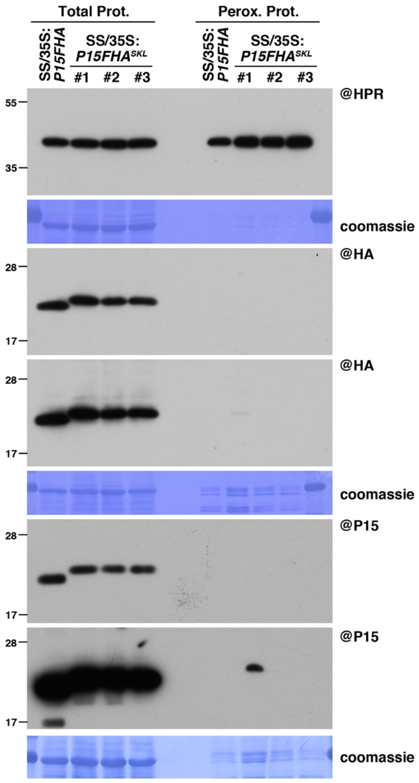

Supplementary Figure 2: (**A**) Western blot analysis of AGO1 and AGO2 accumulation in total and peroxisomal fractions of 35S:P19HA/P19HA^{SKL} transgenic lines on the same samples analyzed in Figure 3D. (**B**) Western blot analysis of P19HA/P19HA^{SKL} accumulation performed with an anti-P19 antibody on the same samples analyzed in Figure 3D. Coomassie staining was used as protein loading control. Figure source data can be found with the Supplementary information.

Supplementary Figure 2

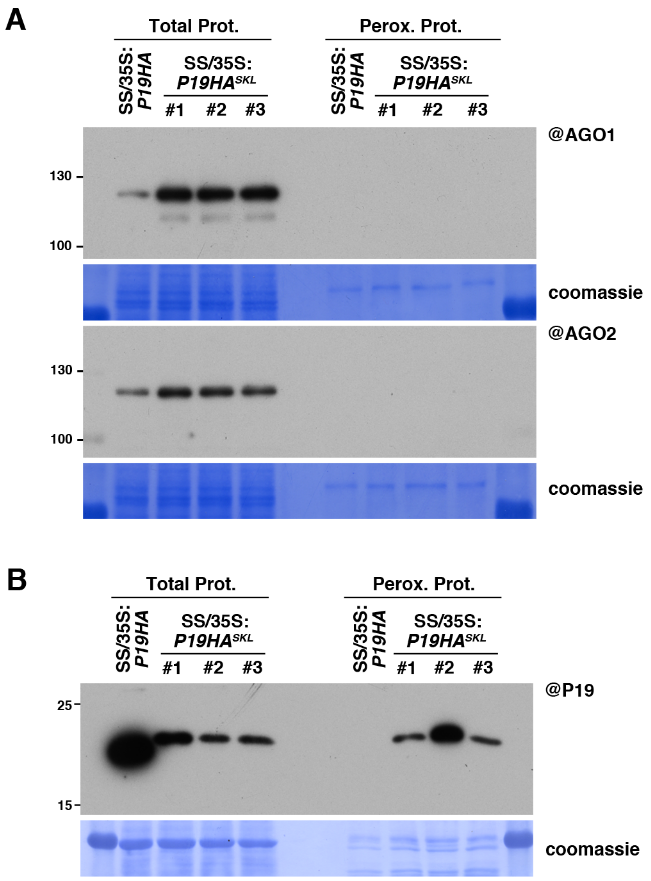

Source Data:

Source Data of Figures 1, 2, 3, 4 and Supplementary Figures 1 and 2.

Source data Figure 1A-B

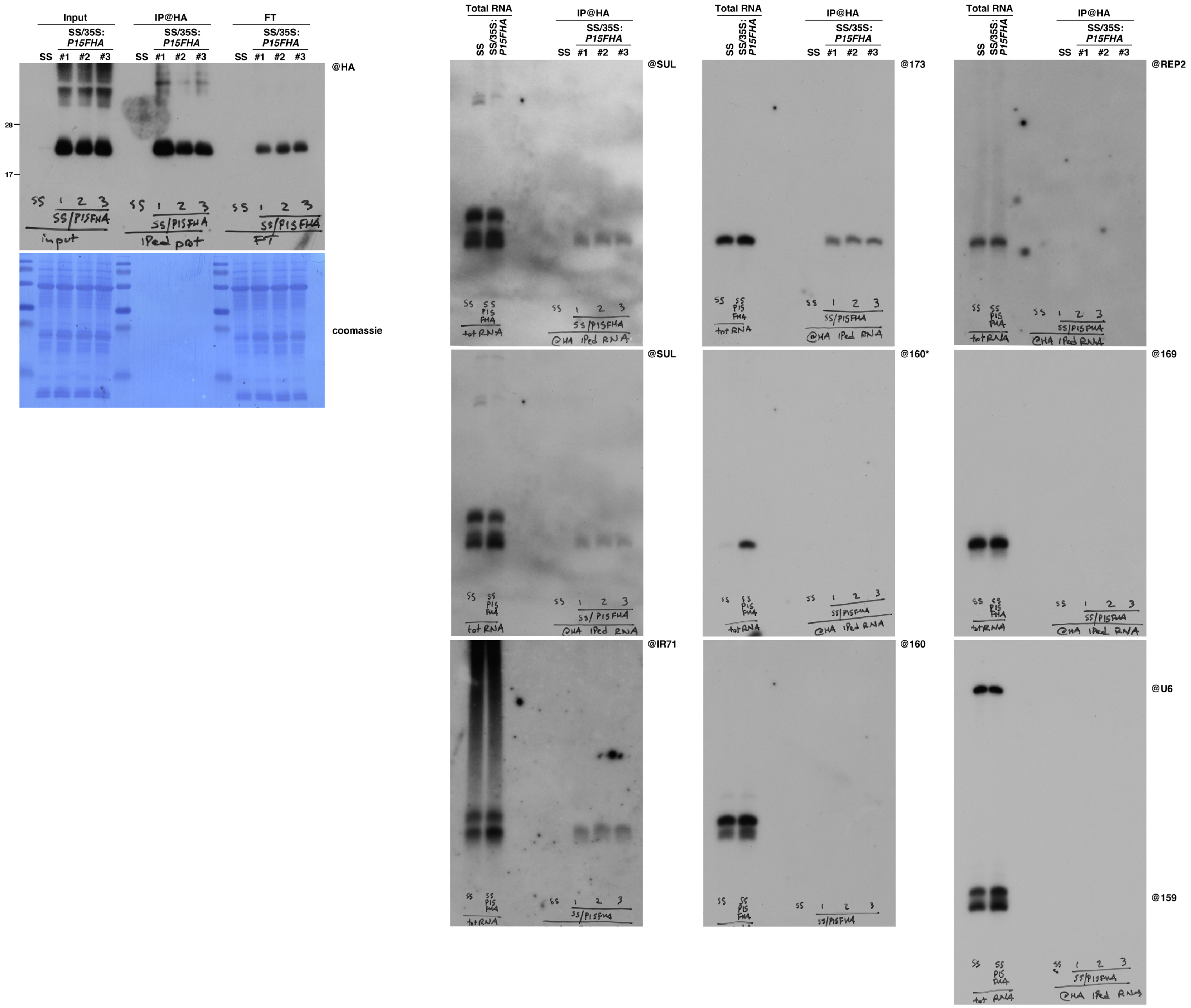

Source data Figure 1C-D and Supplementary Figure 1

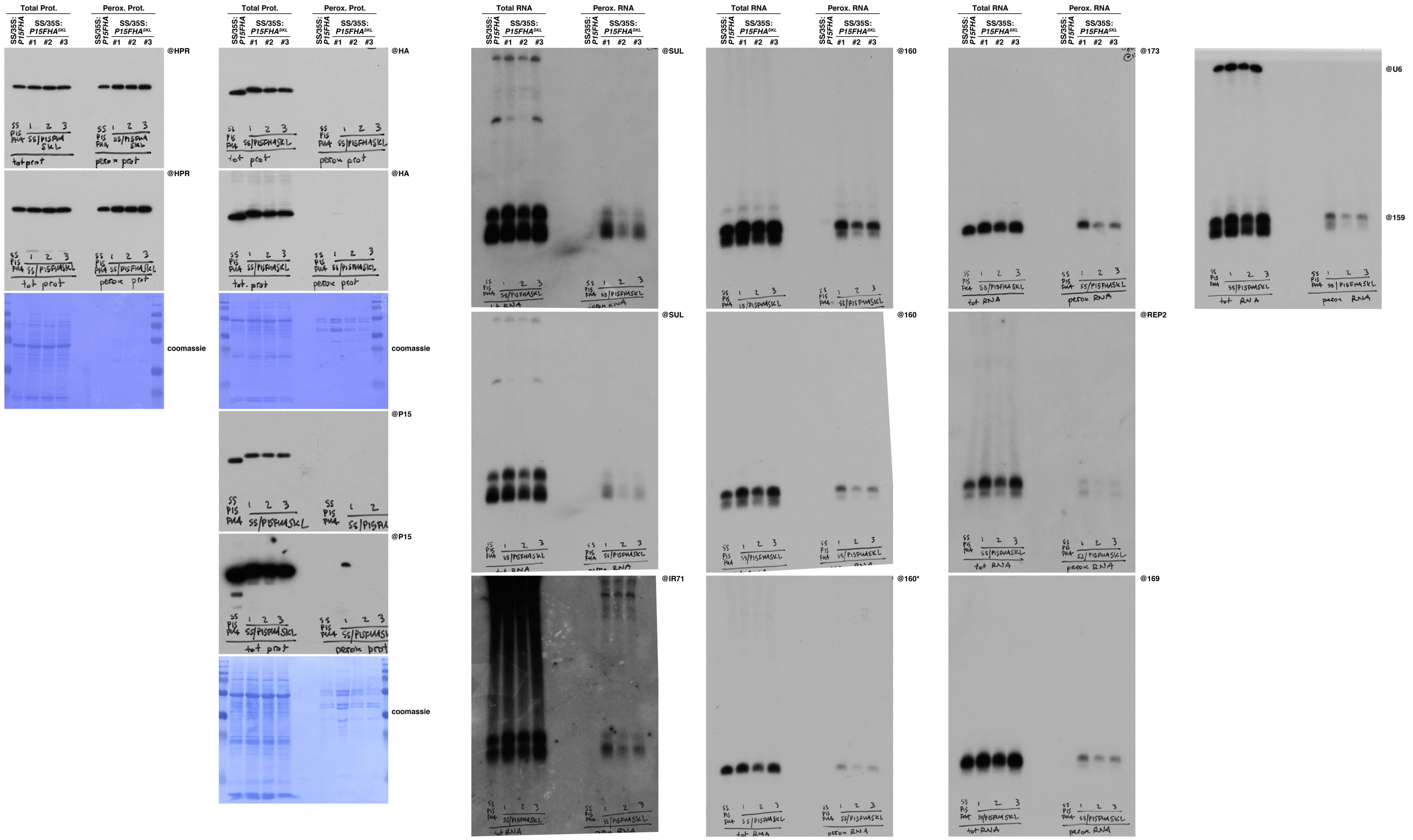

Source data Figure 2

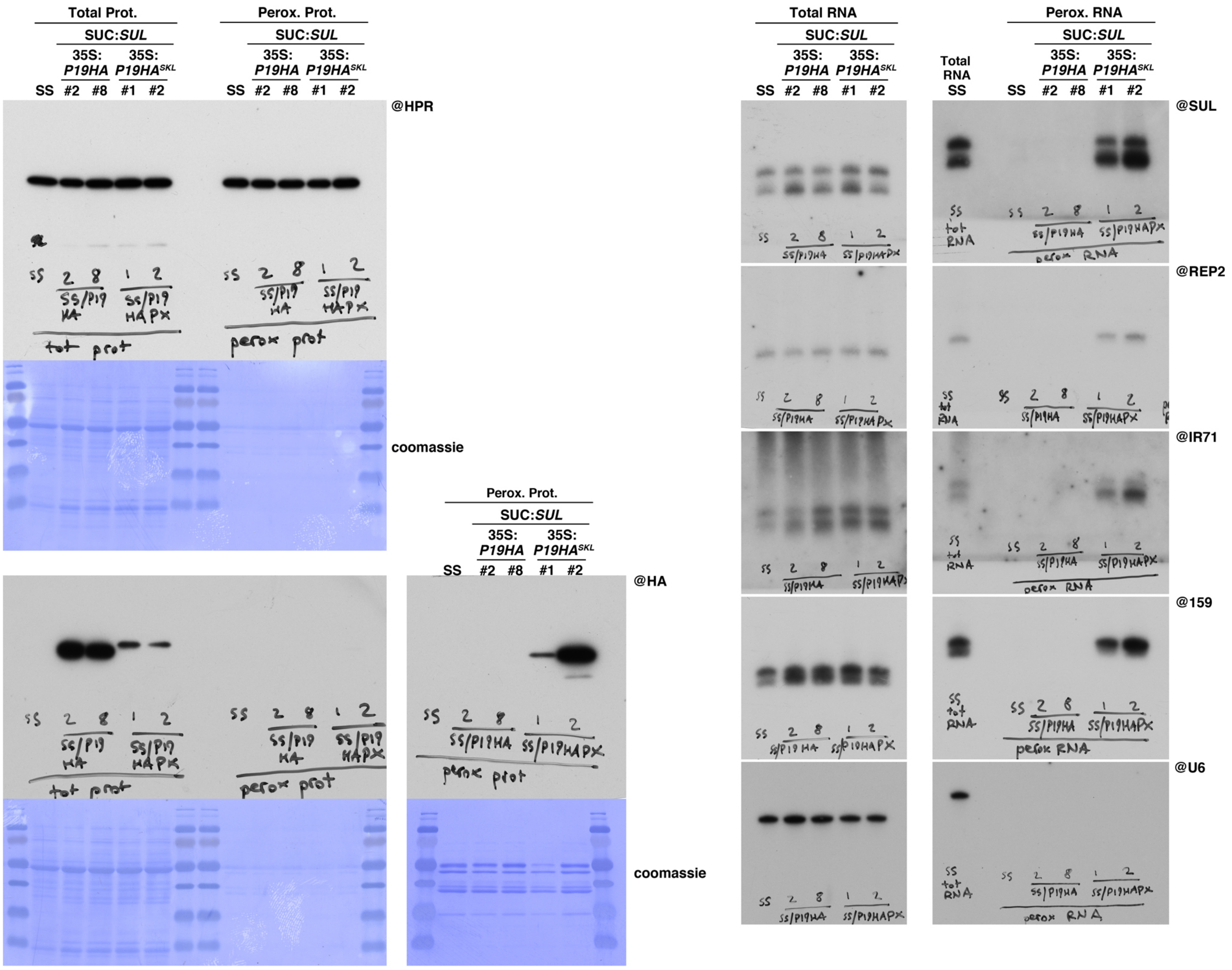

Source data Figure 3A-B

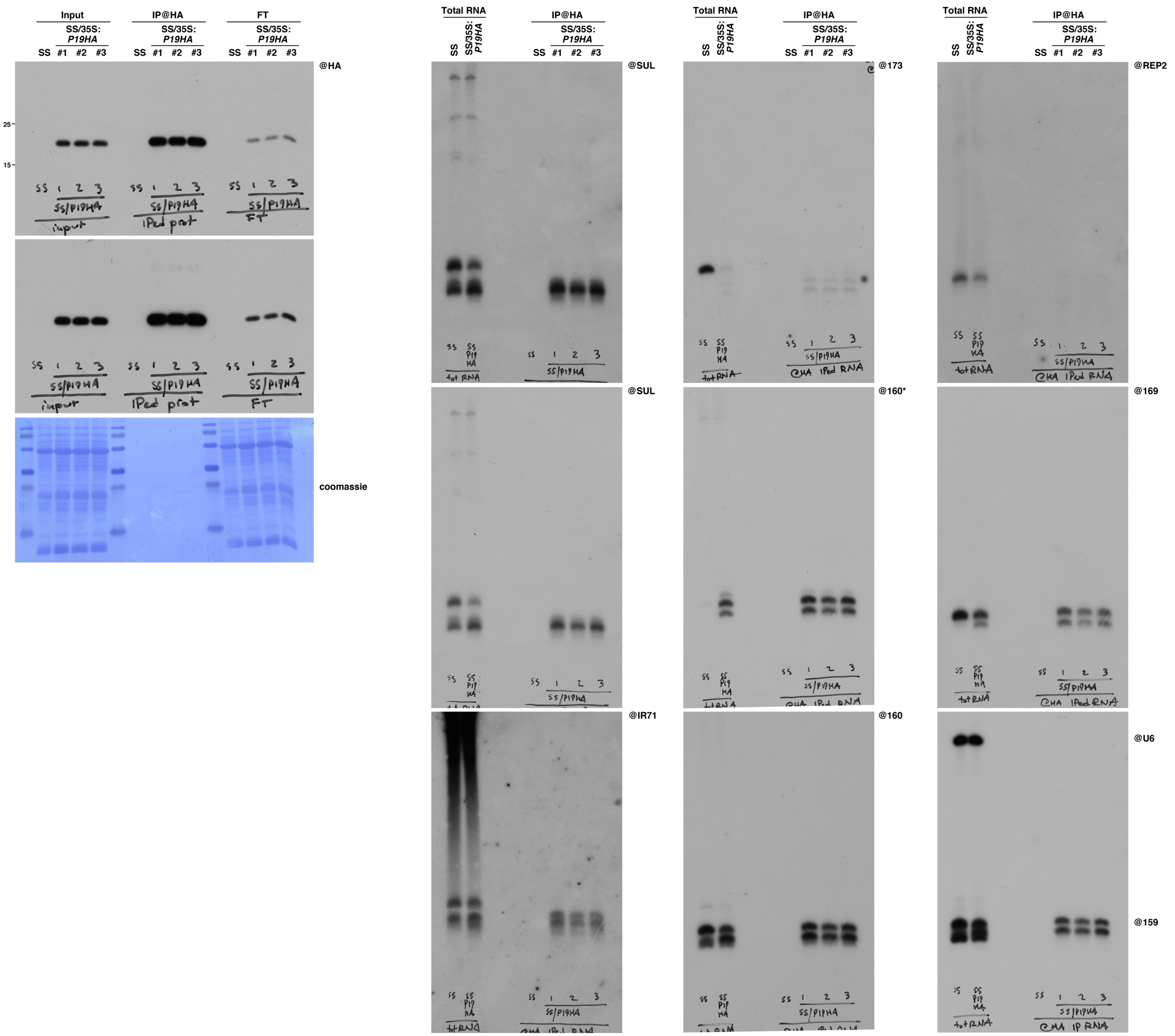

Source data Figure 3C-D and Supplementray Figure 2

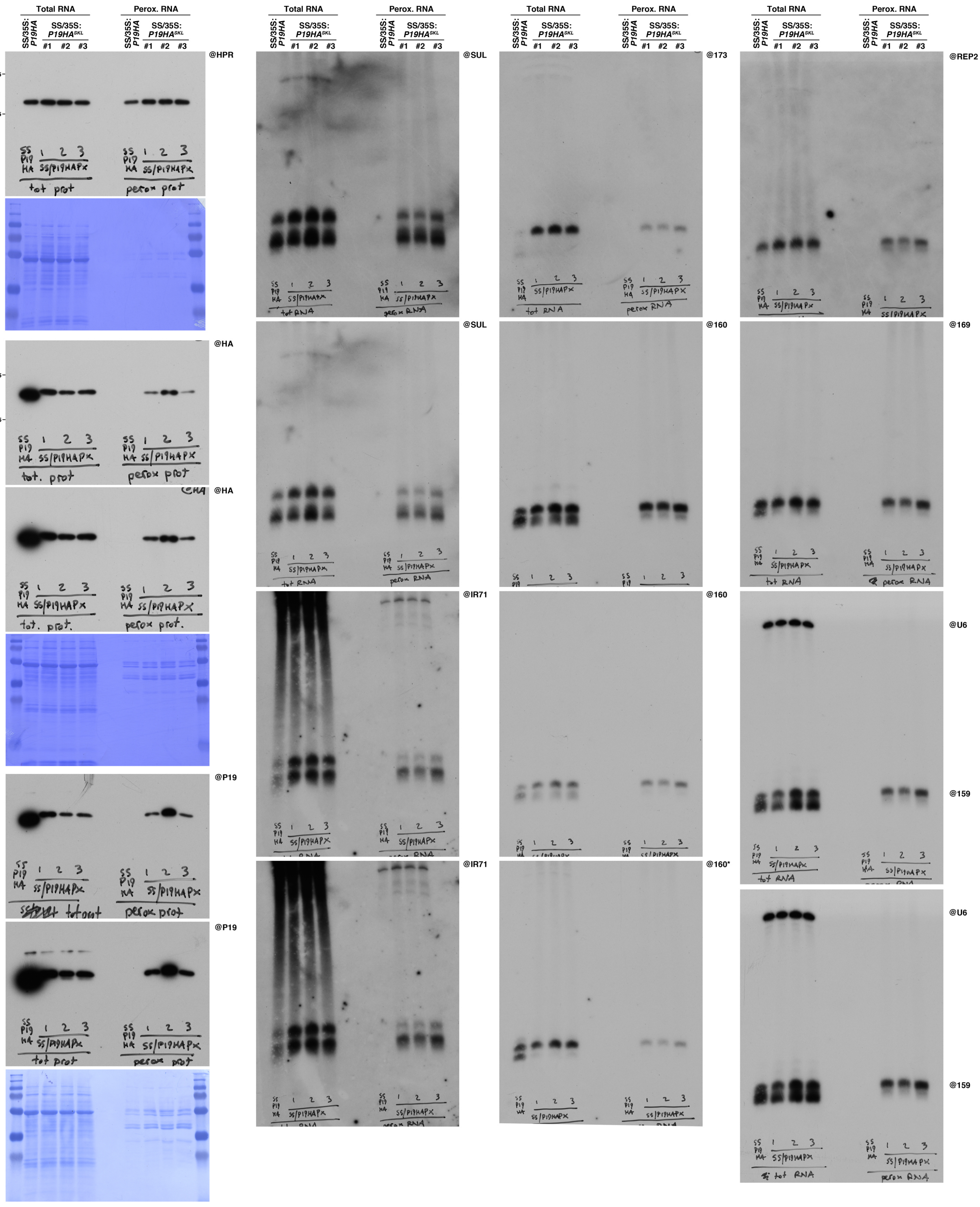

Source Data Supplementary Figure 2

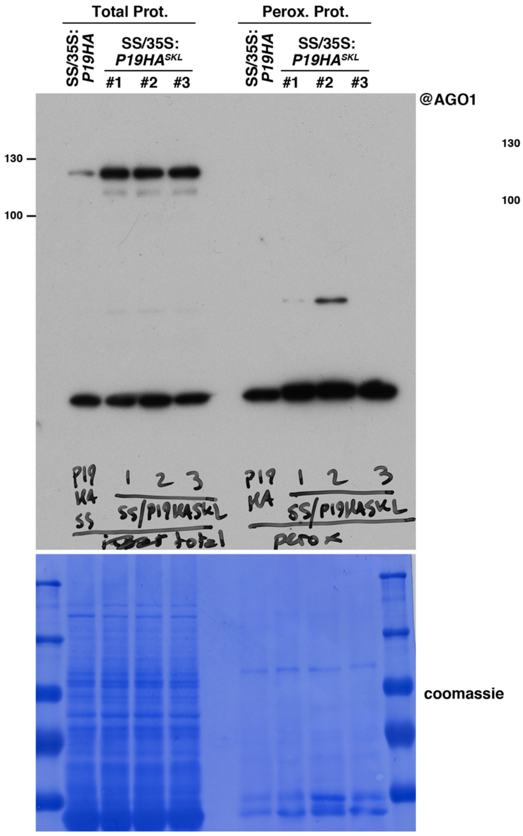

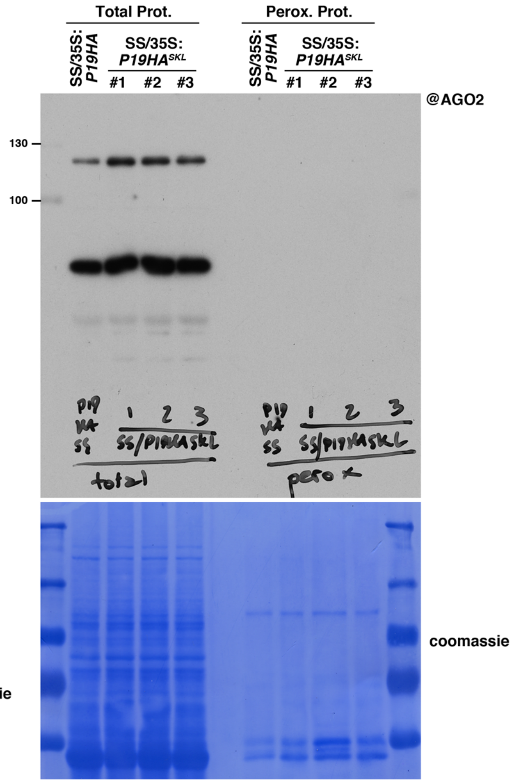

Source data Figure 4

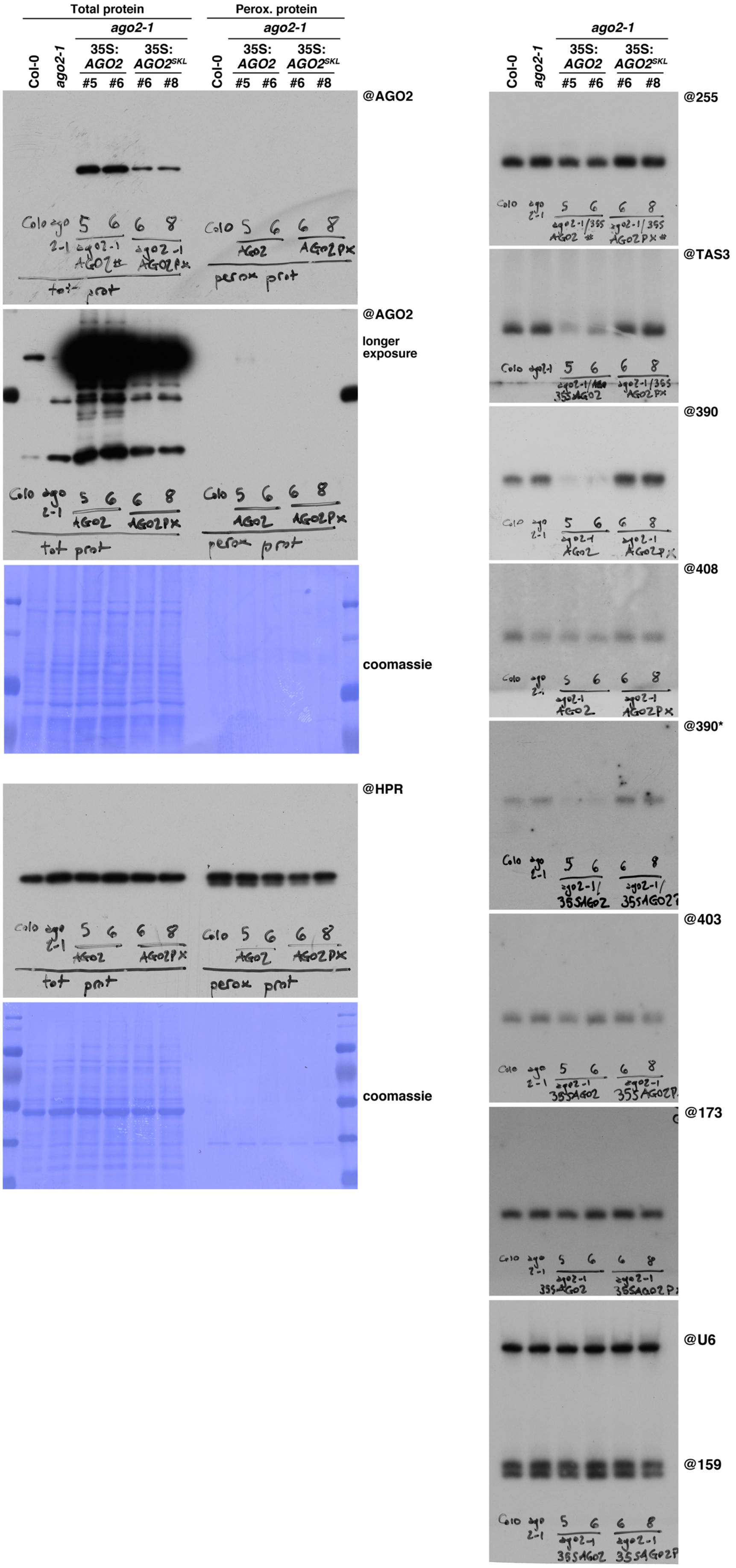

Supplement: Supplementary file 2 [file Image_1.PDF]
